# Complete Genome Sequences of Seven *Vibrio cholerae* Phages Isolated in China

**DOI:** 10.1128/genomeA.01019-17

**Published:** 2017-11-22

**Authors:** Sudhakar G. Bhandare, Andrew Warry, Richard D. Emes, Jingliang Su, Paul A. Barrow, Robert J. Atterbury

**Affiliations:** a University of Nottingham, School of Veterinary Medicine and Science, Sutton Bonington, United Kingdom; b University of Nottingham, Advanced Data Analysis Centre, Sutton Bonington, United Kingdom; c Key Laboratory of Animal Epidemiology and Zoonosis of the Ministry of Agriculture, College of Veterinary Medicine, China Agricultural University, Beijing, China

## Abstract

The complete genome sequences of seven closely related *Vibrio cholerae* phages isolated from environmental sites in southeastern China are reported here. Phages QH, CJY, H1, H2, H3, J2, and J3 are members of the *Podoviridae* family and are highly similar to the previously sequenced *Vibrio* phages VP2, VP5, and phiVC8.

## GENOME ANNOUNCEMENT

Cholera disease is endemic in southeastern China (1), and seven *Vibrio cholerae*-specific phages were isolated from this region. Two phages were isolated from river water in Beijing (QH and CJY), three from separate lakes in Wuhan, Hubei (H1, H2, and H3), and two from a river in Nanchang, Jiangxi (J2 and J3). All seven phages were identified as podoviruses by electron microscopy.

Genomic DNA extraction was done using the Promega A7280 Wizard DNA Clean-Up system, followed by ethanol precipitation (2). Sequencing was done using the Illumina MiSeq platform, generating paired-end 250-bp reads. Reads were assembled using the SPAdes version 3.1.0 assembler (3), with 120× coverage. Phage sequences were assembled into single high-coverage contigs. All of the phages were found to be circularly permuted with terminal redundancy. Annotation was done using Rapid Annotations using Subsystems Technology (RAST) (4), with some manual curation. The predicted protein-coding sequences were compared to known proteins using BLASTp (5), while conserved protein motifs were identified by a Pfam/HHpred search (6). The genomes were scanned for tRNA using tRNAscan-SE (7) and ARAGORN (8).

Of the 7 phages, H1, H2, H3, and J2 have the same genome length and share greater than 99% sequence identity. Phage CJY is also very similar but contains a 12-base insertion (near the N terminus of coding sequence [CDS] CJY_0035) which is also absent in the equivalent CDS of VP2 (GenBank accession no. NC_005879) or VP5 (GenBank accession no. NC_005891). Although they are so similar, these phages were isolated independently from widely separated locations and could be clearly discriminated by restriction fragment length polymorphisms (data not shown). QH and J3 share the presence of an additional 79-amino acid (aa) CDS of unknown function (J3_0046/QH_0045) not present in the other Chinese phages but found in VP2, VP5, and phiVC8. Phage J3 has a 2-base insertion causing a 31-aa C-terminal truncation of CDS J3_0047, due to the usage of an alternate start codon. QH additionally does not contain a short predicted 53-aa CDS of unknown function that is present in the other phages (e.g., H1_0024) and also conserved in VP2, VP5, and phiVC8. Phage QH shows 2 to 3% more sequence divergence from the other Chinese phages, most notably in the N-terminal region of QH_0014 (possible major tail subunit); this may be an example of the mosaic nature of phage genome evolution, as this divergent peptide region is 99% identical to that found in VP5, while in the other phages, the region is 99% identical to that of VP2.

These Chinese phages form a distinct cluster with members of the proposed VP2-like phage group of the *Podoviridae* family (9), VP2, VP5, and phiVC8 (GenBank accession no. JF712866); they share between 86 and 97% identity over 90 to 97% of their lengths with the previously characterized phages. No tRNA sequences were detected in any of the genomes.

### Accession number(s).

This whole-genome shotgun project has been deposited at GenBank (see Table 1). The version described in this paper is the first version.

**TABLE 1:** *Vibrio cholerae* phage genome characteristics

Phage name	GenBank accession no.	Length (bp)	No. of genes	G+C content (%)
QH	KM612259	39,725	48	50.50
CJY	KM612260	39,542	48	50.56
H1	KM612261	39,530	48	50.54
H2	KM612262	39,530	48	50.55
H3	KM612263	39,530	48	50.54
J2	KM612264	39,530	48	50.58
J3	KM612265	39,782	49	50.55
